# Research on occupational health and safety management in the context of big data

**DOI:** 10.3389/fpubh.2024.1514996

**Published:** 2024-12-02

**Authors:** Qianrui Hwang, Min Yao, Shugang Li, Fang Wang, Zheng Li, Tongshuang Liu

**Affiliations:** ^1^College of Safety Science and Engineering, Xi'an University of Science and Technology, Xi’an, China; ^2^State Key Laboratory of High-Efficiency Utilization of Coal and Green Chemical Engineering, Ningxia University, Yinchuan, China; ^3^Ningxia Coal Industry Co., Ltd of China National Energy Group, Ningxia Hui Nationality Autonomous Region, Yinchuan, China

**Keywords:** occupational health and safety management system, big data, occupational disease prevention and control, safety management performance, sustainable development

## Abstract

At present, with the rapid development of China’s economy and industrial transformation, the situation of China’s occupational health and safety is grim, and at this stage, there are still problems such as unsound laws and regulations and standard system, weak awareness of the primary responsibility of the enterprise, weak supervisory, technical capacity, and backward application of information technology. Based on the current situation of occupational health and safety management at home and abroad and relevant theories, this study adopts the method of systematic review and takes the evaluation of China’s occupational health and safety management system as the theme and conducts a valuable exploration of the evaluation research of China’s occupational health and safety management system, analyzes the current situation of occupational health and safety from the aspects of prevention and control of occupational diseases, prevention and control of safety accidents, and monitoring of the psychology of insecurity, and researches the two aspects of governmental regulation and social responsibility It analyzes the current situation of occupational health and safety from the aspects of prevention and control of occupational diseases, prevention and control of safety accidents and monitoring of unsafe psychology, and studies the current situation of post-performance evaluation of the development of OHS management system from the aspects of government supervision and social responsibility, aiming at providing countermeasure suggestions for the sustainable development of OHS management system in China. The research indicates that in the context of the new economic normal, effectively safeguarding workers’ occupational health rights and interests, promoting sustainable and robust economic and social development, as well as enhancing the sustainability of China’s occupational health and safety management system have emerged as challenging yet pivotal areas for comprehensive exploration.

With the continuous development, reform, and transformation of society and economy, “economic transformation” and “sustainable health” have become the themes of the era of economic transformation and development. Protecting workers’ occupational health and safety has become an indispensable guarantee to promote industrial and economic transformation and development. However, the number of occupational diseases, injuries, and deaths remains large. This reflects that occupational health and safety needs to catch up to the pace of industrial economic construction. Occupational health and safety have become the fundamental guarantee for the economic development of all countries, and occupational health and safety management systems have also become an essential part of the economic legislation of many countries in the world. Western developed countries are leading in developing occupational health and safety management system theory and practice exploration. They have established and perfected the OHS management system, including government supervision, and created the achievement of “low or zero accident countries.”

With the transformation and upgrading of China’s economy and the integration of internationalized economic development, China’s occupational health and safety has made significant progress compared to the past. However, a large gap exists between China and Western developed countries. The number of new cases of occupational diseases reported every year is still increasing, which reflects that safety accidents occur from time to time, the implementation of the primary responsibility of enterprises is not in place, the investigation of hidden dangers is only a formality, and the government supervision, especially the grass-roots supervision power and working capacity is relatively weak (1). This shows China’s occupational health management work and occupational hazards are still severe. China is currently undergoing economic transformation, and occupational health and safety management “supporting the transformation and upgrading” has become an important issue of economic development that needs to be resolved. The government, society, and enterprises are increasingly prioritizing the management of occupational health and safety, which can effectively mitigate economic losses resulting from occupational health issues and alleviate pressure on the public healthcare system. This paper analyzes the research related to the safety status of occupational health management from three aspects of occupational diseases, safety accidents, and mental health through the study of domestic and foreign occupational health management methods, combines the research on government control of the performance of occupational health and safety management with the status quo of social responsibility, and puts forward suggestions for the scientific development of China’s occupational health and safety management (2).

## 1 Introduction

The occupational health and safety management system (OHSMS) is a modern safety production management model that emerged internationally in the late 1980s. It is a systematic, procedural, and highly self-restraint, self-improvement scientific management system (3). In the late 1990s, under the influence of the integration of international occupational safety and health standards, the International Organization for Standardization (ISO) tried to develop and expand the occupational safety and health standardized management system so that it would become a standard similar to the ISO9000 and ISO14000 (4).

With the acceleration of trade and investment liberalization, global economic integration worldwide, and the development of technology, the number of occupational accidents and occupational diseases continues to rise in many developing countries (5–7). The rapid growth of occupational diseases in China is mainly due to insufficient awareness of occupational health and safety, inadequate supervision, and one-sided pursuit of economic efficiency (8). As can be seen from Table 1, statistics from China’s Health Planning Commission show that by the end of 2023, there have established total 18,818 healthy enterprises across the country, and have included 185,000 enterprises in the scope of exceptional management of occupational disease hazards, and have carried out extraordinary management work. By the end of 2023, there were 1,297 technical service organizations for occupational health, 666 technical service organizations for radiation health, and 21 organizations for chemical toxicity assessment. A total of 5,670 occupational health inspection institutions reported 21.88 million cases of occupational health inspection, among which 368,000 cases of occupational contraindications and 9,000 cases of suspected occupational diseases were found. 620 occupational disease diagnostic organizations completed 27,740 occupational disease diagnoses. 829 pneumoconiosis rehabilitation stations (points) were built with funds transferred from the central government to local governments, and provided rehabilitation services to 1.2 million people. In 2023, a total of 12,087 new cases of various types of occupational diseases were reported nationwide, among which there were 8,105 cases of occupational pneumoconiosis and other respiratory diseases (among which 8,051 cases were of occupational pneumoconiosis), 2,228 cases of occupational ear, nose, throat, mouth and oral cavity diseases, 625 cases of occupational infections, and 367 cases of occupational chemical poisoning, 577 cases of occupational diseases caused by physical factors, 65 cases of occupational skin diseases, 67 cases of occupational tumors, 39 cases of occupational eye diseases (including 1 case of radioactive cataract), 7 cases of occupational radiological diseases, and 7 cases of other occupational diseases (9). Regarding industry distribution, occupational disease cases are mainly concentrated in the mining, manufacturing, metallurgy, and building materials industries.

**Table 1:** Statistics of new cases of occupational diseases reported nationwide.

Types of occupational diseases	Number of new cases						
	2017	2018	2019	2020	2021	2022	2023
Occupational pneumoconiosis	22,701	19,468	15,898	14,367	11,809	7,577	8,051
Other respiratory diseases	89	56	49	41	68	38	54
Occupational ENT and oral diseases	1,608	1,528	1,623	1,310	2,123	1879	2,228
Occupational infectious disease	673	540	578	488	339	308	625
Occupational chemical poisoning	1,021	1,333	778	486	567	399	367
Occupational diseases caused by physical factors	399	331	264	217	283	749	577
Occupational dermatosis	83	93	72	63	83	10	65
Occupational tumor	85	77	87	48	79	71	67
Occupational eye disease	70	47	53	24	43	23	39
Occupational radiation sickness	15	17	15	10	5	11	7
Other occupational diseases	12	7	11	10	8	5	7
Total person	26,756	23,497	15,898	17,064	15,407	11,108	12,087

It is easy to see that China’s occupational health and safety situation is still severe and restricts China’s economic development. In the current transition stage of economic development, with the gradual improvement of the level of safety management and the emergence of new safety management concepts and methods, the key issues and research direction of China’s occupational health and safety management system have shifted to improving the modern safety management level of enterprises and promoting a systematic and scientific occupational health and safety system in China.

## 2 Methodology

This study adopted the systematic review method in literature retrieval. Web of science (Web of Science Core Collection) and CNKI are chosen as the database in this study, as they cover a wide range of national and international journals. Search attributes and their values are presented in Table 2.

**Table 2:** Literature search methods.

Search attributes	Values used in the search
Database	Web of science(Web of Science Core Collection) or CNKI
Keywords	“Occupational health and safety” or “occupational health and safety management performance”
Search scope	Topic
Year published	From 2004 to present
Document type	Journal article
Language	English or Chinese

The scope of this study is limited to the research and application of occupational health and safety, as well as the performance of occupational health and safety management. Firstly, it focuses on a few relevant subject fields such as engineering, medicine, social science, and psychology. Secondly, only journal articles are considered for inclusion in this study; specifically, research papers, papers, and review papers are retained after preliminary review while book reviews and editorials are excluded. Based on the initial search results, a systematic review approach is adopted for further analysis by selecting journals related to occupational health and safety science. The secondary search process not only emphasizes the study of occupational health and safety management systems but also takes into account their current practical applications.

## 3 Literature review and current status of occupational health and safety in the context of big data

**Figure 1:**
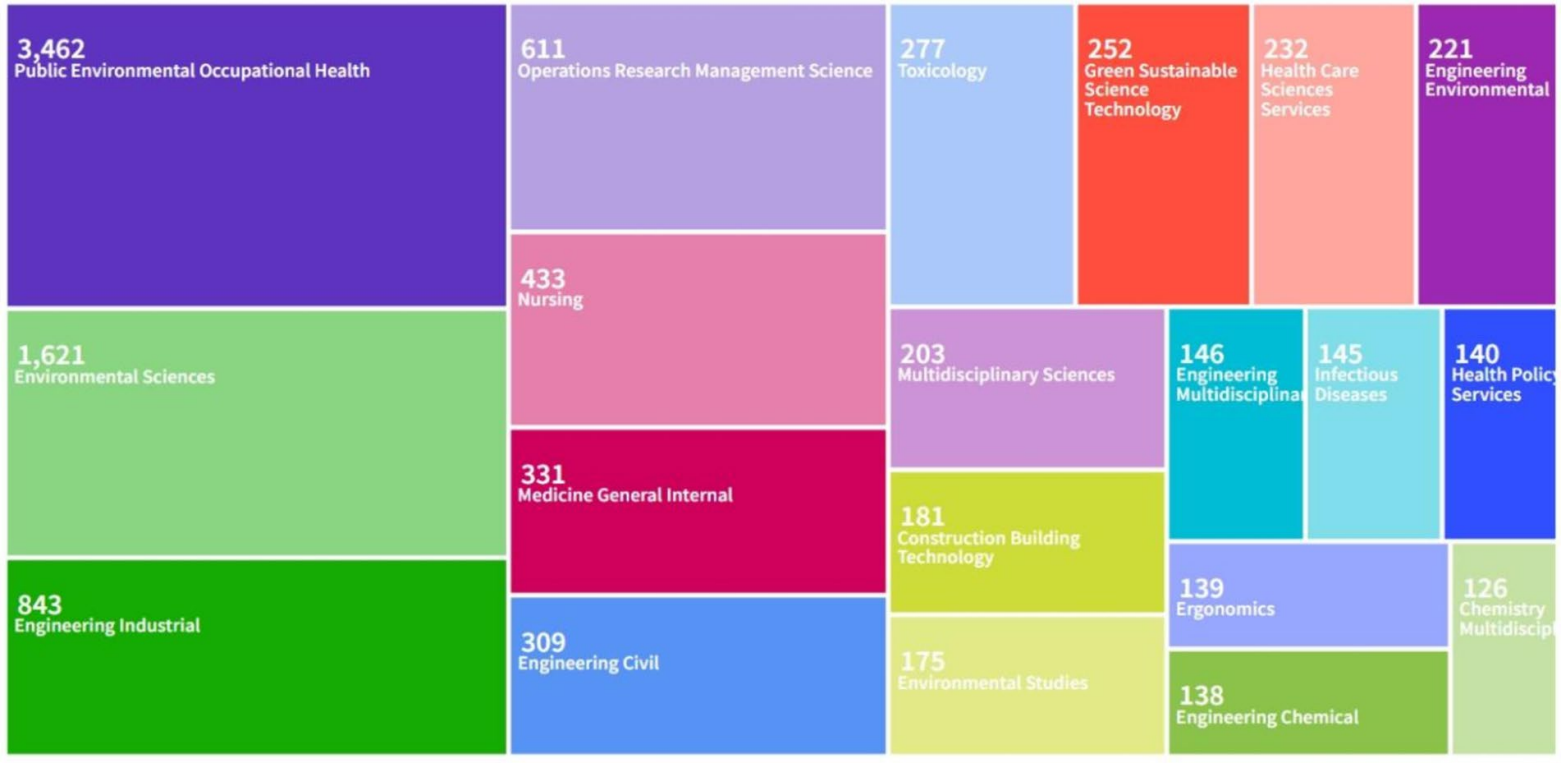
Paper main topic distribution diagram on web of science.

**Figure 2:**
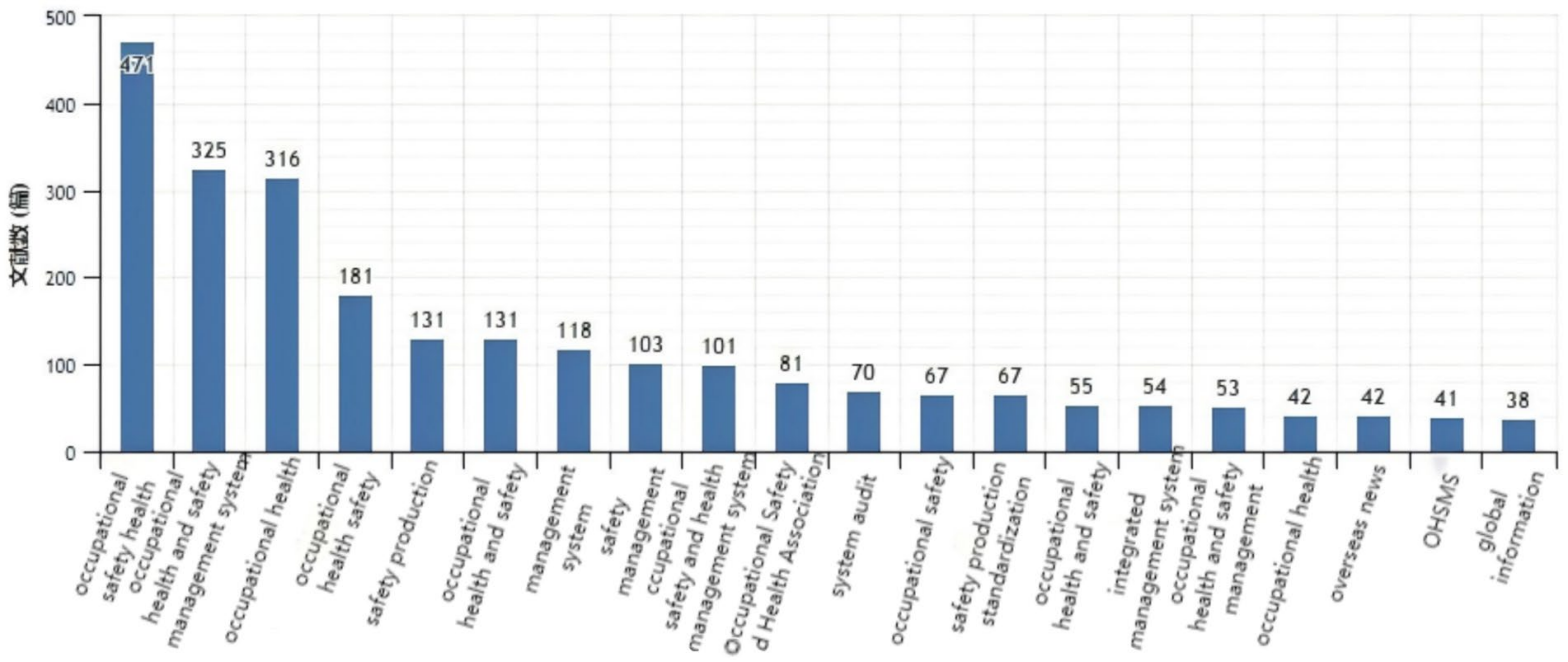
Paper main topic distribution diagram on CNKI.

**Figure 3:**
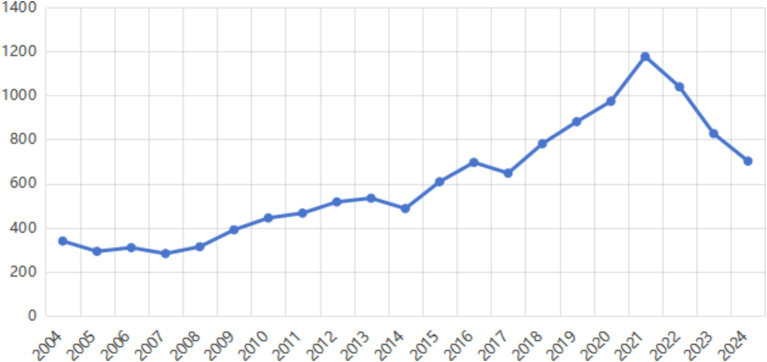
The fluctuation in literary output from 2004 to 2024.

By searching the journal papers with the topic of “occupational health and safety” through web of science (Web of Science Core Collection) and CNKI from 2004 to 2024, there are 8,443 and 4,262 related papers were retrieved on web of science (Web of Science Core Collection) and CNKI, respectively. As shown in Figures 1, 2, the visual analysis of literature research on occupational health and safety shows that the research topics mainly focus on production safety, occupational health and occupational health. As shown in Figure 3, the research literature on occupational health and safety, both domestically and internationally, demonstrates a discernible upward trend, indicating the growing emphasis placed on this field in current times.

### 3.1 Current status and research on occupational diseases

Most research on occupational diseases in foreign countries focuses on industrial activities such as small and medium-sized enterprises. The categorization of occupational diseases varies from one industry to another, such as in the construction industry, where most of the occupational diseases are caused by employees suffering from shoulder pains, back pains, skin-related illnesses, problems with the eyes, and respiratory and noise irritation problems (10). Meanwhile, in small and medium-sized industries, it is found that the work-related occupational disease injuries suffered are different due to gender, monthly salary, age, work experience, and personal use (11). In addition, physical occupational hazards are positively associated with muscle, bone, or joint pain. The higher the exposure to physical hazards such as physical fatigue, adverse environmental factors, and repetitive exercise, the greater the likelihood of workers developing occupational diseases (12). Therefore, regulating occupational diseases requires official participation in managing the disease, as well as that of associations and civil organizations, which have highly dynamic forms of regulation and multifaceted regulatory content (13).

Domestic, occupational disease research is mainly focused on the industrial and mining industries with the highest incidence of occupational diseases, and the occupational diseases that are prone to incidence are dust-induced pneumoconiosis, noise-induced noise deafness, and toxic and hazardous gases-induced leukodystrophy. Therefore, combining safety assessment and occupational disease management will be the new direction of improvement. This new direction can improve the ability to manage preventive occupational disease and compose a new evaluation system of occupational health and safety management in order to improve the overall level of occupational health and safety management in China (14). In controlling and supervising occupational diseases, the government should take the primary responsibility for relief. In contrast, individual citizens’ relief and public welfare organizations’ relief are indispensable forces, and the focus of employees suffering from pneumoconiosis to fight for their rights and interests is their own individual awareness and group self-help (15).

### 3.2 Current status and research on security incidents

The accident causation chain is based on the view that all safety accidents are based on unsafe actions caused by human management negligence, and this view has become the typical representative of the accident causation chain. 1990, the “Swiss cheese” model view that the root causes of accidents are a combination of individuals, behaviors, and social relationships triggered by accidents has become the representative of the modern accident causation chain. This view has since become the modern representation of the accident causal chain (16). From the perspective of occupational safety climate, the correlation between safety climate and accidents in Denmark has been analyzed using a cross-adjusted regression model, which shows a gradual correlation between the number of safety climate problems and the ratio of accidents.

The research on domestic safety accidents started late and primarily focuses on industrial and mining industries where the most safety accidents occur. For coal mine safety accidents, the mathematical method of FAHP can be used to process the indicators of four aspects: mining personnel, machinery and equipment, working environment, and safety conditions, and to construct a safety early warning system for coal mines (17). Moreover, in order to prevent coal mine safety accidents, we should focus on reality and establish a long-term effective mechanism to find the root causes behind the safety accidents from the four aspects of people, machinery, environment, and management so as to provide strong evidence for the solution to the safety accidents (18, 19). The “2–4” model suggests that the root cause of accidents is the lack of an organization’s safety management system, based on an analysis of China’s current safety situation, individual behavior, and organizational factors (20).

### 3.3 Current status and research on mental health

In China, less attention has been paid to research on occupational mental health, the most common being the implementation and evaluation of EAP (Employee Assistance Program). The International Labor Office’s research on this theory laid the foundation for this field, and this research is by far the most comprehensive research foundation. In addition to occupational and mental health, the ILO has also actively explored safety climate, job burnout, work stress, work environment, and other aspects. With the advancement of relevant research, it has been observed that due to unfavorable working conditions and a higher likelihood of accidents, workers exhibit lower levels of mental well-being compared to the general population. Consequently, psychological issues such as occupational stress resulting in tension, anxiety, depression, and other related problems have progressively emerged as significant concerns within the field of occupational health both domestically and internationally (21–23).

Mental health is a personal characteristic that can influence our behavior. It depends on the environment and personalities within and outside work and is related to job content, conditions, employment, and working relationships. The International Labor Office (ILO) 2000 introduced the concept of short-term mental health (e.g., mood and emotions, behaviors such as apathy, physiological reactions, etc.) and long-term mental disorders (e.g., exhaustion, post-traumatic stress disorder, depression, psychosis, cognitive disorders, drug use, etc.), arguing that in times of economic recession special attention needs to be given to the management of health and safety activities that occur in workplaces that are preventive of the mental health of the employees of a company (24). The concept of traumatic stress disorder is a critical component of the concept of mental health.

Research in the area of occupational health and mental health in China has been concerned with a relatively late period of time, and the breadth and depth of the areas covered have been limited. Usually, researchers establish models of psychological elements (situational, defective, and endogenous) and, based on obtaining relevant data, analyze the influence relationship between unsafe psychological elements and such models according to the established safety psychometric models. Through the system dynamics model, the simulation of group factors and other influencing factors toward the coal mine employees’ safety and mental health level of intervention strength, based on the service content and service mode of EAP (Employee Assistance Program), can form the best path to improve the level of employees’ safety and mental health and the best intervention application strategy (25).

## 4 Literature review and current status of OHS management performance in the context of big data

**Figure 4:**
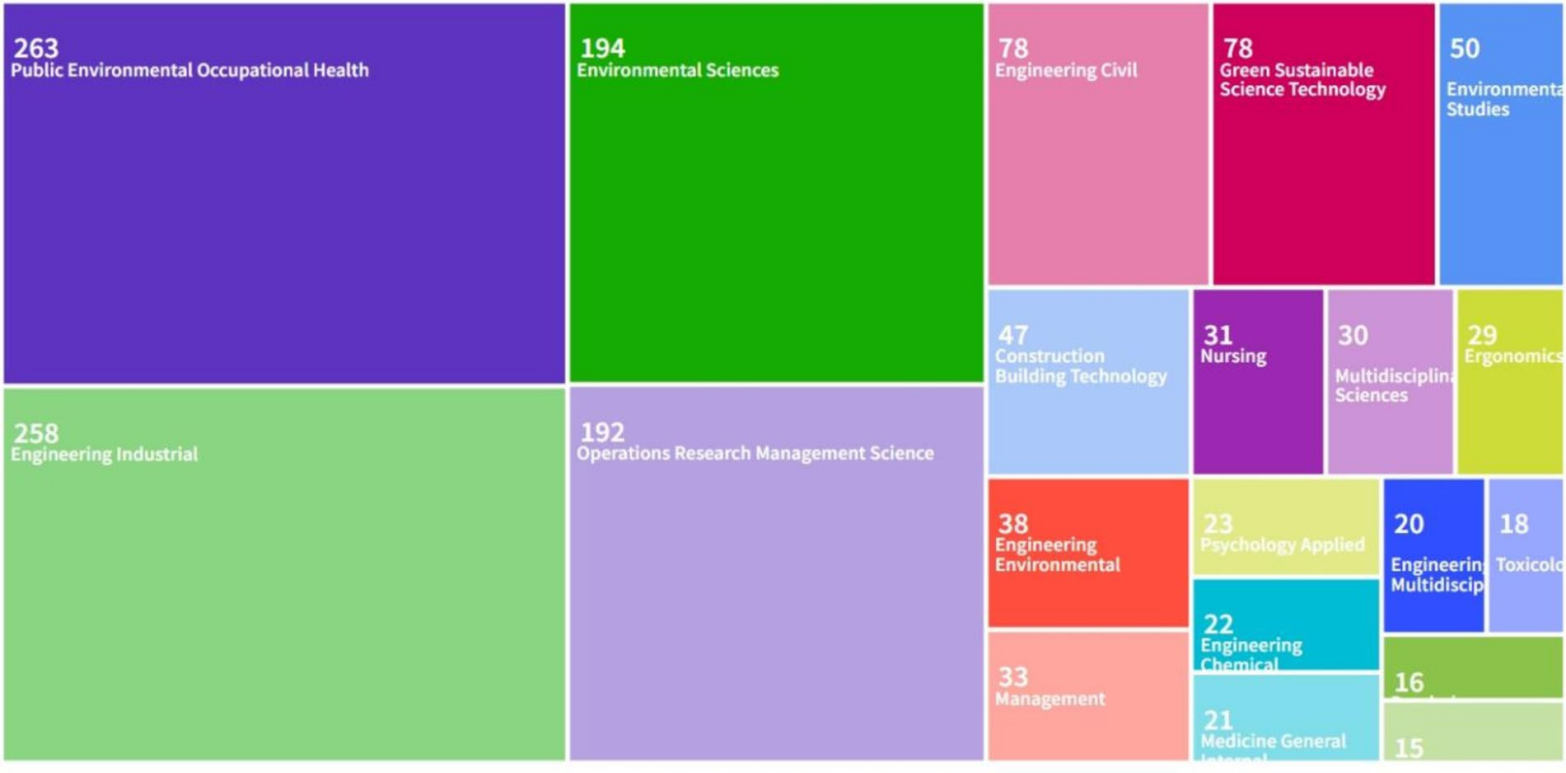
Paper main topic distribution diagram on web of science.

**Figure 5:**
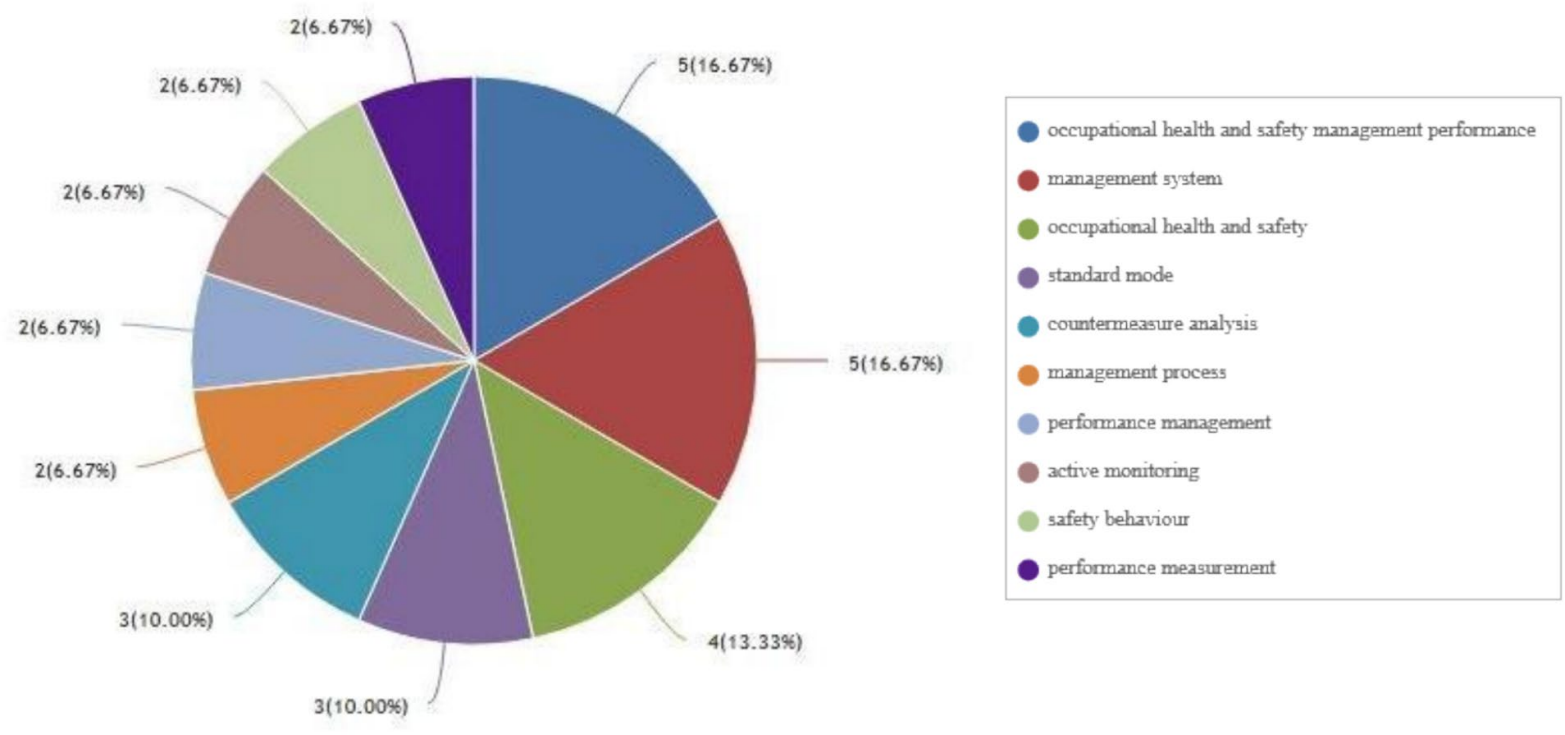
Paper main topic distribution diagram on CNKI.

**Figure 6:**
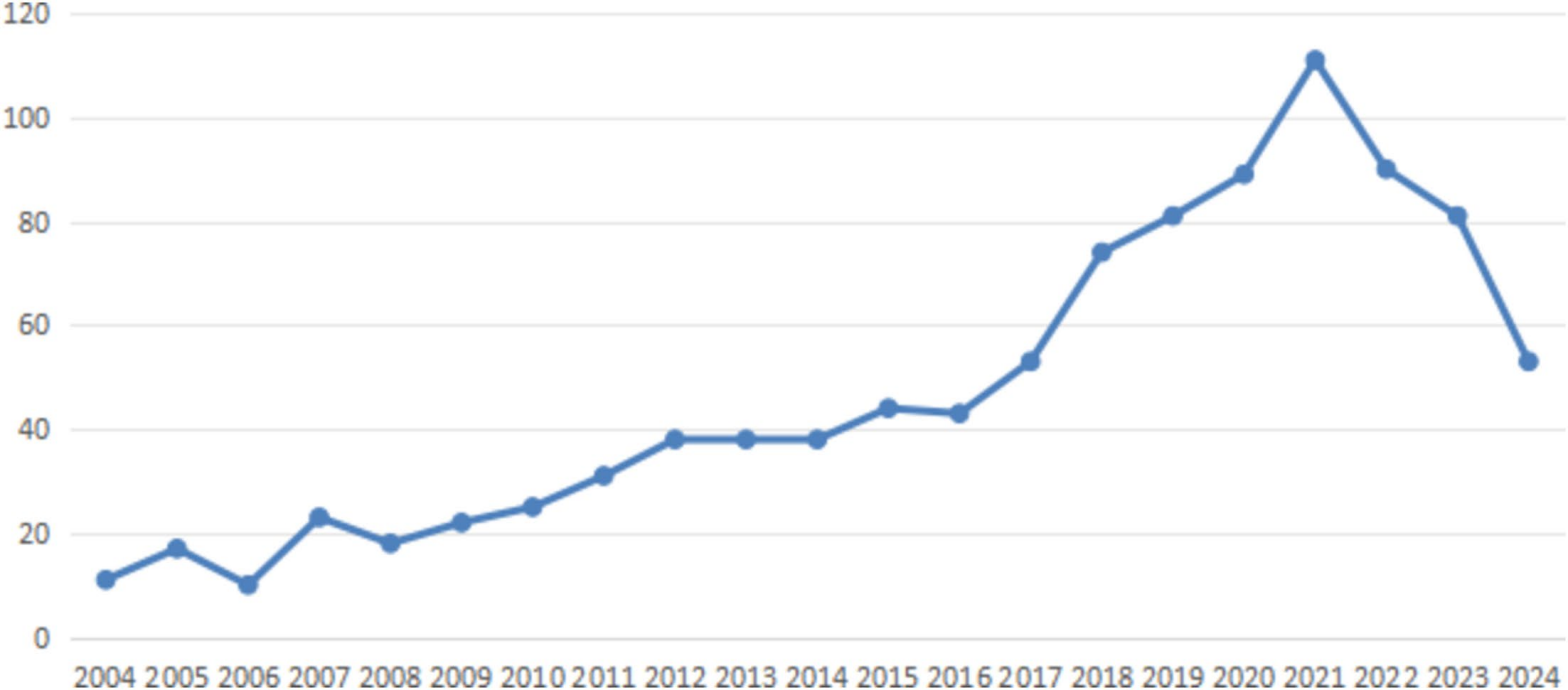
The fluctuation in literary output from 2004 to 2024.

By searching the journal papers with the topic of “occupational health and safety management performance” through web of science (Web of Science Core Collection) and CNKI from 2004 to 2024, there are 938 and 52 related papers were retrieved on web of science (Web of Science Core Collection) and CNKI, respectively. As shown in Figures 4, 5, the visual analysis of literature research on occupational health and safety shows that the research topics mainly focus on management system, standard mode, countermeasure analysis and management process. As shown in Figure 6, research literature on occupational health and safety management performance at home and abroad mainly shows an increasing trend but decreased after 2021. There is increasing attention on occupational health and safety management, and the research field categories are increasingly subdivided.

### 4.1 Current status and research on government regulation

For government control research earlier, foreign developed countries formed a theoretical system. Although these studies emphasize the effect of control, continuous adjustment and revision have formed a more complete system that can be adapted to the respective national conditions. In the United States, the central national occupational health regulatory agencies are the Occupational Safety and Health Administration (OSHA), the Mine Safety and Health Administration (MSHA), and the National Institute for Occupational Safety and Health (NIOSH) (26). The United Kingdom pioneered the Industrial Revolution, and poor working conditions led to an epidemic of occupational and infectious diseases. Therefore, in the 19th century, Britain enacted some laws such as the Factories Act, the Mines Act, the Public Health Act, and the Employers’ Liability Act to protect workers’ occupational health (27). In Germany, a highly industrialized country, occupational health management began in the 1850s. Because Germany has a large proportion of industrial workers, it is characterized by a dual model of occupational health management with government regulation and industry associations (28). The manufacturing industry in the United States between 1979 and 1998 is used as a case study to analyze examples of government regulation of occupational health and safety in the United States, and the results show that government regulation can be an excellent way to promote the performance of occupational health and safety management (29). An essential theme in developing the OHSAS regulatory system, “State-funded inspectorates to enforce and promote worker participation in the OHS legal system,” traces the legal regulation of health, worker and other people’s safety (30).

The research on domestic government regulation is based on analyzing countermeasures for coal mine safety regulations. Aiming at the series of problems of government regulation of coal mines in China, the study analyzes some problems and reasons of coal mine safety in government regulation. Therefore, the government regulation of safety in China’s coal mining industry should strengthen the law enforcement, the self-discipline of enterprises, the construction of safety culture and the social publicity mechanism (31, 32). Moreover, China should strengthen its people-oriented management and realize the essential safety management of enterprises in the long-term mechanism of government supervision (33).

### 4.2 Current status and research on social responsibility

OHS social responsibility involves an overview of OHS management, relevant employment information, welfare and health facilities, health education, accident statistics, OHS management and safety. The practical implementation of an OHS management system can be significantly improved by applying innovative OHS management systems that have been effectively implemented and measured in the EU, together with appropriate incentives.

At present, more studies at home and abroad are on whether corporate social responsibility (CSR) is positively correlated with financial performance, and they are mainly distributed in industrial and mining industries. The establishment of a mining enterprise social responsibility index evaluation system is of great significance for solving the problems of mining enterprises and carrying out research on mining enterprise social responsibility. The more influential international index systems for measuring CSR are the Dow Jones Sustainability Index (DJSI), Domini Socially Responsible Investment Index (KLD), Global Reporting Initiative (GRI), and Social Responsibility Guide (IS026000). We should further study and apply these index systems and continue to explore the CSR application mechanism, certification system development model, etc., suitable for China’s OHS social responsibility (34). Based on studying the content of mining CSR and stakeholder relations, we should construct an evaluation index system to effectively solve China’s OHS management problems, such as environmental and labor issues (35). The fulfillment of CSR by enterprises should also establish a research framework suitable for CSR and values and drives suitable for China’s economic characteristics and CSR, and pay attention to entrepreneurship, strategic research and development, etc. (36)

## 5 Discussion and illumination

Existing domestic and international literature provides new perspectives for studying occupational health and safety management systems. Domestic research on the contents of the OHS management system started late and is mainly based on the industrial and mining industries where safety accidents are frequent and the incidence of occupational diseases is high. Since there are fewer studies on other industries, this also requires us to study and evaluate the contents of OHSMS comprehensively. Due to the low awareness of governmental control by the managers of some business units and the insufficient investment by enterprises in the working environment, occupational health protection, and health check-ups of their employees, new safety problems and occupational disease hazards will continue to emerge with the wide application of new technologies, processes, equipment, and materials. This also poses new challenges to preventing and controlling occupational diseases and managing occupational health and safety. However, in foreign countries, as shown in Table 3, the regulatory aspects of OHS management include law enforcement regulation by government departments, assistance from relevant associations, and autonomous regulation by civil organizations. This is a highly dynamic form of regulation. For example, in developed countries such as the United Kingdom, Germany, and the United States, in terms of occupational health and safety management, although they emphasize the framework of occupational health and safety management system, to a certain extent, it is also consistent with the development of each country’s economy, politics, culture and so on. The more economically developed countries are, the more importance they attach to managing safety accidents and preventing occupational diseases, which has resulted in outstanding performance, such as the world-famous domino model.

**Table 3:** Comparison of OHS between China and foreign countries.

Comparison content	Difference			
	China	Australia	Germany	Britain and America
Way	Volunteer	Volunteer	Force	Force
Supervise	Social regulation	The government and insurance agencies jointly supervise	The government and insurance agencies jointly supervise	The Work Environment Authority
Scope	Health and safety is mainly reflected in the body of employees	Health and safety are mainly reflected in the physical and psychological aspects of employees	Health and safety are mainly reflected in the physical and psychological aspects of employees	Health and safety are mainly reflected in the physical and psychological aspects of employees
Laws and regulations	Reflected in the overall framework	Reflected in the specific framework, and the enforcement	Reflected in the specific framework, and the enforcement	Reflected in the specific framework, and the enforcement
Educational training	Yes	Take education and training as the fundamental measure to prevent accidents	Take education and training as the fundamental measure to prevent accidents	Take education and training as the fundamental measure to prevent accidents
Role of industrial injury insurance institutions	Work-related injury compensation	Work consultation and supervision, work hazard investigation, analysis, testing, employee physical examination, formulation of relevant system documents, etc.	Work consultation and supervision, work hazard investigation, analysis, testing, employee physical examination, formulation of relevant system documents, etc.	Work consultation and supervision, work hazard investigation, analysis, testing, employee physical examination, formulation of relevant system documents, etc.
Health monitoring	Way of third-party supervision	Government and the insurance structure forced	The government forced	The government forced
Performance appraisal	Yes	Clear and definite	Clear and definite	Clear and definite

Based on this, China’s OHS management system’s operational status and management performance are comprehensively evaluated by drawing on domestic and international OHS management system-related research contents, perspectives, evaluation methods, etc. In implementing and developing the OHS management system in China, we should focus on the following aspects: Firstly, continuously improve the legal and regulatory system. The occupational health legislation of the United States, the United Kingdom and Germany was completed in the 1970s, and has been perfected after nearly half a century of continuous revision and development. China’s occupational health laws and regulations are still at the stage of exploration and revision, especially the revision of laws and standards, which should be clearly defined as the central unit and department responsible, with deadlines set and the frequency of updating increased. The perfect bill not only is guarantee for the safety and health of workers, but also can make the overall management of occupational health more effective. Then, strengthen the government’s supervision and monitoring efforts. In Sweden, occupational health and safety management is mandatory. In Germany and Australia, the government is multi-channel, large-scale supervision of the implementation of occupational health and safety management, and are actively involved in vocational skills training, accident investigation and other aspects. However, our government supervision of occupational health and safety management is minimal; only in the enterprise of more severe accidents does the role of government supervision appear. Therefore, China should moderately increase the strength of the government supervision and punishment, meanwhile, special inspect the duplication of problems to ensure that the scientific nature of supervision and monitoring means diversity, and ultimately enhance the importance of the enterprise to the management of occupational health and safety. Thirdly, strengthen the use of information technology. Informatization means should occupy an essential position in studying occupational health and safety management systems. Using extensive data collection to statistically analyze the occupational health and safety management data of industries and enterprises and form reports can effectively help the government and associations and provide strong support for diagnostic evaluation and early warning measures. Strengthen the interoperability and sharing of occupational health data among all relevant departments and establish a system for workers to lay the foundation for future informatization, data-based statistical analysis, and scientific research. Improve the occupational health and safety management system and realize the balanced and sustainable development of economic, environmental, and social benefits brought by the occupational health and safety management system (37).

## 6 Conclusion

In recent years, although the overall situation of occupational safety and health management in China has stabilized and improved compared with the past, safety accidents still occur from time to time. China is currently in a period of economic transition, which will inevitably bring about occupational health problems. By drawing on domestic and international research and development status, we will improve the occupational health and safety management system regarding government control and social responsibility in China. In a period of rapid industrial development and reform, this can better prevent safety accidents and combat occupational diseases and workers’ mental health conditions at the root. Sustainable development emphasizes holistic development, which promotes economic development and coordinates the balanced development of the environment, society, and other aspects. Whether it is the comparative analysis of economic benefits and evaluation of environmental benefits in foreign countries or the perspective of sustainable development in China, we must treat economic, social, and environmental benefits holistically and systematically. Therefore, improving occupational health and safety management at this stage in China requires more social attention so that more workers can be aware of such issues. Supervisory authorities should also use more scientific and diverse methods to improve the quality of work so that enterprises can implement the primary responsibility to truly achieve the results of the occupational health-related level of protection of workers as the core of the enhancement of the level of occupational health because the adequate protection of the occupational health of employees is far greater than the pursuit of profit maximization brought about by the economic, social and environmental benefits (38, 39).

## Data Availability

The original contributions presented in the study are included in the article/supplementary material, further inquiries can be directed to the corresponding authors.
